# Estimating the Growing Stem Volume of Coniferous Plantations Based on Random Forest Using an Optimized Variable Selection Method

**DOI:** 10.3390/s20247248

**Published:** 2020-12-17

**Authors:** Fugen Jiang, Mykola Kutia, Arbi J. Sarkissian, Hui Lin, Jiangping Long, Hua Sun, Guangxing Wang

**Affiliations:** 1 Research Center of Forestry Remote Sensing and Information Engineering, Central South University of Forestry and Technology, Changsha 410004, China; jiangkriging@csuft.edu.cn (F.J.); linhui@csuft.edu.cn (H.L.); longjiangping@csuft.edu.cn (J.L.); gxwang@siu.edu (G.W.); 2 Key Laboratory of Forestry Remote Sensing Based Big Data and Ecological Security for Hunan Province, Changsha 410004, China; 3 Key Laboratory of National Forestry and Grassland Administration on Forest Resources Management and Monitoring in Southern Area, Changsha 410004, China; 4 Bangor College China, Bangor University, 498 Shaoshan Rd., Changsha 410004, China; m.kutia@bangor.ac.uk (M.K.); a.sarkissian@bangor.ac.uk (A.J.S.); 5 Department of Geography and Environmental Resources, Southern Illinois University, Carbondale, IL 62901, USA

**Keywords:** forest growing stem volume, coniferous plantations, variable selection, texture feature, random forest, red-edge band

## Abstract

Forest growing stem volume (GSV) reflects the richness of forest resources as well as the quality of forest ecosystems. Remote sensing technology enables robust and efficient GSV estimation as it greatly reduces the survey time and cost while facilitating periodic monitoring. Given its red edge bands and a short revisit time period, Sentinel-2 images were selected for the GSV estimation in Wangyedian forest farm, Inner Mongolia, China. The variable combination was shown to significantly affect the accuracy of the estimation model. After extracting spectral variables, texture features, and topographic factors, a stepwise random forest (SRF) method was proposed to select variable combinations and establish random forest regressions (RFR) for GSV estimation. The linear stepwise regression (LSR), Boruta, Variable Selection Using Random Forests (VSURF), and random forest (RF) methods were then used as references for comparison with the proposed SRF for selection of predictors and GSV estimation. Combined with the observed GSV data and the Sentinel-2 images, the distributions of GSV were generated by the RFR models with the variable combinations determined by the LSR, RF, Boruta, VSURF, and SRF. The results show that the texture features of Sentinel-2’s red edge bands can significantly improve the accuracy of GSV estimation. The SRF method can effectively select the optimal variable combination, and the SRF-based model results in the highest estimation accuracy with the decreases of relative root mean square error by 16.4%, 14.4%, 16.3%, and 10.6% compared with those from the LSR-, RF-, Boruta-, and VSURF-based models, respectively. The GSV distribution generated by the SRF-based model matched that of the field observations well. The results of this study are expected to provide a reference for GSV estimation of coniferous plantations.

## 1. Introduction

Forest plays a crucial role in the global carbon cycle as one of the largest carbon sinks in the biosphere [1,2]. Estimating forest growth and productivity is, therefore, essential for informing climate change research and forest management efforts globally [3]. Forest growing stem volume (GSV) refers to the total volume of tree trunks in a given forested area that reflects the richness of forest resources and the health of the forest ecosystem. Accurate estimation of the GSV, therefore, plays an important role in both forest resource management and the monitoring of ecosystem dynamics [4,5,6]. Compared with traditional field surveys, remote sensing technology allows for a more efficient approach to the monitoring and management of forest resources in real-time [7,8,9]. However, the accuracy of GSV estimation using remotely sensed data is determined together by data sources, feature variable selection methods, and estimation models.

Extracting information on vegetation growth from remote sensing data is the basis of forest parameter estimation. The common remote sensing data sources for GSV estimation are varied in terms of active and passive remote sensing data. Optical data is a typical passive remote sensing data that is derived from multi-spatial resolution and multi-spectral sensors, which provides ground feature information as a range of different wavelength bands. For example, MODIS, SPOT-VEGETATION, NOAA/AVHRR, FY-3/MERSI [10,11,12,13] are common optical datasets used for GSV estimation at global and regional scales. Hyper-spectral data, such as those from Hyperion [14], AVIRIS [15] and HYDICE [16], can effectively detect the characteristics of ground objects. Hyper-spectral data contains nearly continuous spectral information, which greatly improves the recognition of different objects on the land surface. Low-resolution sensor data having high temporal resolution and wide spatial coverage enables the analysis of time-series characteristics of vegetation at large scales. However, a large number of spectral bands and information redundancy of hyper-spectral data lead to huge computational loads. With the enhancement of remote sensing technology, a large number of datasets with higher spatial resolution have been recently used for GSV estimation. Medium and high-resolution data, such as those provided by Landsat [17] and GaoFen (GF) [18] systems, can obtain more accurate vegetation attributes. Using these data, the mixed pixels are reduced, thus significantly improving the mapping of vegetation parameters. However, due to cloud coverage, data stripe, long satellite revisit periods, and high data costs in acquiring data, it is often difficult to obtain sufficient satellite data covering a given target area.

Active remote sensing refers to the transmitting of electromagnetic waves to a detected target area and receiving the echo signal of an object [19], mainly including light detection and ranging (LiDAR) and synthetic aperture radar (SAR). LiDAR can provide three-dimensional structural information efficiently and has unique advantages in estimating tree height and spatial structure. Among LiDAR datasets, airborne LiDAR is the most effective but also the most expensive. SAR can provide observations under all weather conditions and is not affected by atmospheric propagation [20,21]. However, SAR signals are influenced by the terrain, and the terrain effects cannot be completely corrected [22,23]. LiDAR and SAR offer three-dimensional point cloud data to record the location and characteristics of a target object in detail. But due to massive data processing requirements, there are limitations in using LiDAR and SAR for acquiring vegetation information at large scales.

The Sentinel-2 carries a multi-spectral imager (MSI) for land monitoring, providing vegetation, soil, and water cover. The red-edge bands have priority over other spectral variables in modeling. The vegetation indices composed of reflectance of the red-edge bands are highly correlated with GSV, which can effectively improve the estimation accuracy of the structural parameters of the planted forests [24,25,26]. Currently, Sentinel-2 is the only source of freely available optical data that exceeds three red-edge bands. Sentinel-2 data can, therefore, provide a more cost-effective potential for accurately mapping GSV of forest plantations [27]. Compared with MODIS and Landsat, the Sentinel-2 data has a higher spatial resolution, which can obtain more accurate vegetation information. Its four 10 m resolution bands and six 20 m resolution bands get much more accurate vegetation growth status in forests. In addition, Sentinel-2 data characterizes higher temporal resolution (revisit period of five days using 2A and 2B). The time-series data provided by the satellite system allow us to obtain high-quality records of seasonal forest changes for forest resources monitoring [27].

Feature variable combination is extremely important for model development and prediction of forest GSV. In the process of modeling, using a large number of independent variables or predictors often leads to complex models and overfitting. Moreover, as the number of predictors increases, the prediction accuracy of GSV from the models may not necessarily increase and, in turn, may decrease due to the increased noise and errors of the input variables. Before modeling, it is thus critical to select the predictors that significantly contribute to improving the estimation accuracy, increasing the interpretability of models, and reducing the running time of the models [28,29]. Pearson correlation coefficient combined with linear stepwise regression (LSR) is commonly used for selecting feature variables, which are linearly related [30]. However, due to the complexity of forest ecosystems, the linear relationship may limit the estimation accuracy of models [27]. The importance assessment of random forest (RF) provides a metric for judging the contributions of feature variables to increasing model prediction accuracy, which can offer an evaluation of non-linear relationships between feature variables and GSV. RF can provide the importance weights of individual variables, but it lacks the ability to directly determine the optimal combination of feature variables. The same feature variable in different combinations of predictors may have different contributions to reducing the errors of models. There have been a few studies on variable selection using random forest associated importance, such as Boruta and Variable Selection Using Random Forests (VSURF), but the effect of these methods in forest estimation is limited [31]. Moreover, different combinations of feature variables may lead to different overall contributions to the improvement of models. The existing methods for the selection of feature variables lack the ability to determine an optimal combination of feature variables. Furthermore, the existing methods may take much time to complete in the large data sets available. In fact, the primary reason why many existing methods are established is to improve accuracy, and the runtime is often hard to balance [31]. There is a strong need to improve the existing methods or to develop a new method that can be used to determine an optimal and stable combination of feature variables for modeling [32].

Presently, remote sensing images or laser point clouds are usually combined with the field measurements to establish models for regional GSV estimation by parametric or non-parametric methods [33,34]. The parametric methods are easy to understand and fast to learn, but they cannot quickly retrieve the correct objective function form in complex conditions [35]. Non-parametric methods do not make strong assumptions about the form of objective functions and are more suitable for the prediction of complex data [33,34,35,36,37,38]. As a representative of non-parametric methods, RF has become popular for forest GSV estimation [36,37]. RF is an ensemble learning method that is insensitive to noise data and does not require any assumptions about the distribution of input datasets. It estimates forest GSV by swiftly constructing a large number of regression trees. During training, the regression trees are independent of each other, and the training speed is rapid. In addition, RF can evaluate the importance of each feature variable in the model, which can effectively judge the contribution of individual variables to the model [38].

This study aims to establish a random forest regression (RFR) model for GSV estimation of coniferous plantations through developing a novel feature variable selection method based on importance evaluation and analyzing its accuracy and effectiveness. In order to verify the application of the modified feature variable selection method, Sentinel-2 and the observed GSV data of Wangyedian Forest Farm were combined with mapping the GSV in the study area. Moreover, four widely used feature variable selection methods (LSR, RF, Boruta, and VSURF) were used and analyzed for comparison. In addition, the effect of texture features in the red-edge bands on the improved GSV mapping was also studied.

## 2. Materials and Methods

### 2.1. General Description of the Study Area

**Figure 1:**
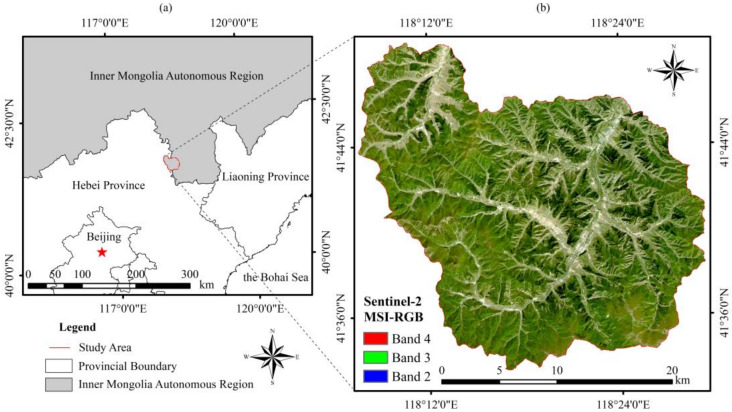
(**a**) Location and boundary of the Wangyedian forest farm and (**b**) Sentinel-2 image covering the study area.

The Wangyedian forest farm is located in Harqin (longitude 118°09′–118°30′ E, latitude 41°21′–41°39′ N), Inner Mongolia Autonomous Region, China (Figure 1). The altitude ranges from 500 to 1890 m. The forest farm has a mid-temperate continental monsoon climate with an annual average temperature of 4.2 °C, a frost-free period of 117 days, average annual sunshine of 2913.3 h, and average annual precipitation of 400 mm. The total area of this region is 25,958 ha, with a forested area of 23,118 ha. The total volume of living trees is 1.28 million m^3^. The dominant tree species are Chinese pine (*Pinus tabuliformis*) and larch (*Larix principis-rupprechtii* and *Larix ologensis*).

### 2.2. Sampling Design and GSV Survey

**Figure 2:**
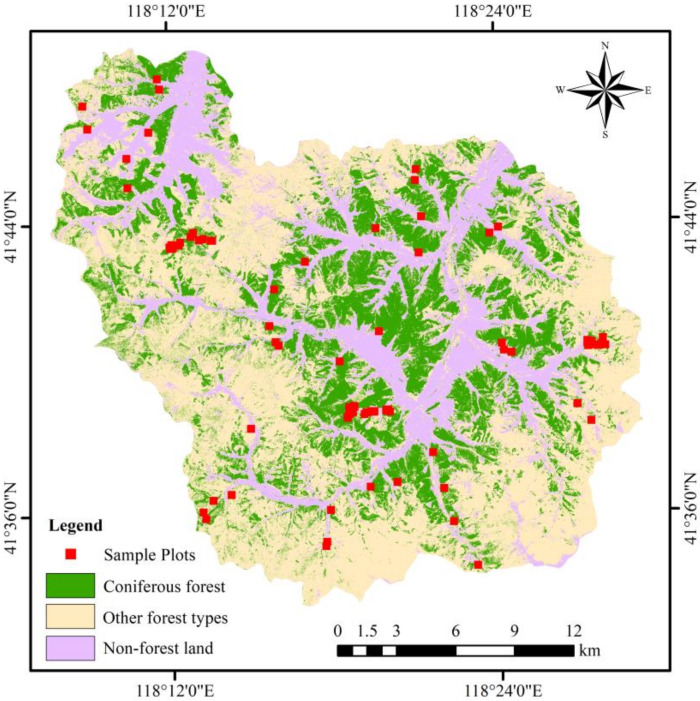
Land-cover types in the Wangyedian forest farm and the spatial distribution of sampled plots.

According to the forest survey data of the Wangyedian forest farm in 2017, the land types were divided into (1) coniferous forest; (2) other forest types (broadleaf forests, coniferous, and broad-leaved mixed forests); and (3) non-forest land (farmland, buildings, water, and unused land). The species, distribution, and coverage range of coniferous forests were analyzed and sorted. The statistical results show that the planted coniferous forests consisted of mainly Chinese pine and Larch. The boundary between the two tree species was determined, and random sampling was used to select 81 sample plots of 25 m × 25 m from the coniferous forests in the study area (Figure 2).

The field survey was carried out from 20 September to 15 October 2017. Trimble Geo 7x Global Positioning System (GPS) was used to record the central coordinates of each sample plot. The forest compass was used to determine the boundaries of the sample plots. Tree height, diameter at breast height (DBH), and environmental factors (air temperature and soil moisture) in each sample plot were measured. Trees in the sample plots with DBH greater than 5 cm were selected and examined. The GSV of each tree was determined using tree height and DBH based volume formula stated by the National Forestry and Grassland Administration of China (http://www.forestry.gov.cn/). The plot-level GSV value of each sample plot was obtained by summing the tree volumes within each plot and then converted to the hectare-level. The GSV values in the study area range from 86.17 to 514.96 m^3^/ha. The mean value, standard deviation, and coefficient of variation of all the sample plots are 209.01 m^3^/ha, 119.87 m^3^/ha, and 44.2%, respectively. At the significant level of 0.05, the confidence interval for GSV is from 209.55 to 254.92 m^3^/ha (Table 1).

### 2.3. Remote Sensing Data and Preprocessing

Two Sentinel-2 multi-spectral images were downloaded as the Level-1C product covering the whole study area from the scientific data hub (https://scihub.copernicus.eu/). These images were acquired during the field investigation time on 22 September, 2017 (Table 2). The official Sen2cor module version 2.5.5 was used to transform the Level-1C product into the Level-2A product [27]. The Level-2A product is the bottom-of-atmosphere corrected reflectance after radiometric calibration and atmospheric correction. Sentinel-2 has 13 bands with three resolutions [26]. Four 10 m spatial resolution and six 20 m spatial resolution bands were used in this study to extract spectral information (Table 3). The cubic convolution interpolation method was used to resample the selected Sentinel-2 bands in order to match the pixel size with the sample plot size and acquire accurate vegetation information.

### 2.4. Extraction and Selection of Spectral Variables and Topographic Factors

Spectral variables are the basis of GSV modeling and mapping. An appropriate spectral variable combination can markedly improve the accuracy and efficiency of modeling [39,40]. Each forest parameter has different reflection characteristics in different bands. Vegetation indices (VIs) were obtained by combining the bands, which can be used to quantitatively describe the vegetation condition. VIs have been widely used in vegetation coverage monitoring and forest parameter estimation. Additionally, the red-edge vegetation index can accurately reveal vegetation health, which correlates closely with the GSV [41,42,43]. Topographic factors are significantly correlated with forest cover in mountainous regions. Slope, aspect, and elevation are topographic factors commonly used. These factors are extracted from the Digital Elevation Model (DEM) raster data in the study area. Twenty feature variables were derived from the Sentinel-2 images and the DEM covering the Wangyedian forest farm, including ten multi-spectral bands, seven VIs (four common VIs and three red-edge VIs), and three topographic factors (Table 4).

**Figure 3:**
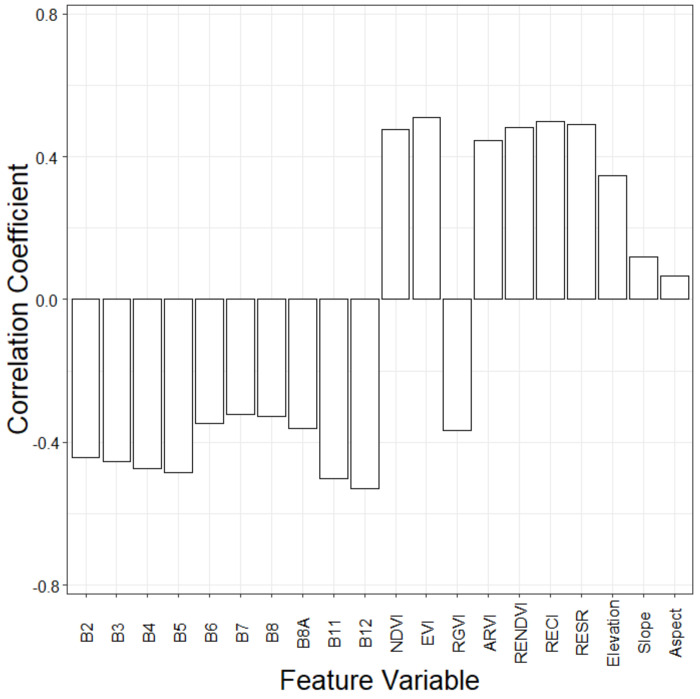
Correlation coefficients of the extracted feature variables with GSV.

The correlation coefficient reflects the strength of the relationship between variables. Pearson correlation coefficient was used to measure the relationship between GSV and the 20 feature variables (Figure 3). Eighteen feature variables significantly related to the GSV were selected for subsequent modeling. Overall, the Pearson correlation coefficients between VIs and GSV were greater than those of the original bands and topographic factors. The feature variables with higher correlation coefficients mainly come from the combinations of red-edge and near-infrared bands.

A complex combination of feature variables leads to low modeling efficiency, resulting in exceeded calculations and suboptimal accuracy of estimation. Therefore, the feature variables that contribute most to reducing the error of a model are first selected to form variable combinations, which are then used to build the model. The appropriate combination of variables can greatly shorten the running time of the model, improve the accuracy of estimation and the interpretability of the model [38]. In order to explore the influence of combinations of feature variables on modeling, feature variables were selected to form the combinations by five methods, including LSR, RF, Boruta, VSURF, and stepwise random forest (SRF) that was proposed in this study. The feature variable combinations selected by the five methods were then used for developing GSV estimation models.

The LSR requires feature variables with a high correlation with GSV to be used in the screening process. The feature variables were introduced into the model, and the significance test was carried out one by one. Statistically significant variables (*p*-value < 0.05) in line with the range were retained to form the final variable combination in the LSR [47]. For each variable combination, the collinearity between the feature variables was detected to avoid the estimation distortion of the model. The variance inflation factor (VIF) was used to measure the collinearity between feature variables, and the threshold value of the VIF was set at 5. The feature variable combination formed by LSR consisted of B7, Elevation, Red-green vegetation index (RGVI), and Red Edge Normalized Difference Vegetation index (RENDVI). The statistical results of the LSR showed that all the selected variables had significant correlations with GSV, and there was no obvious collinearity between the variables (Table 5).

**Figure 4:**
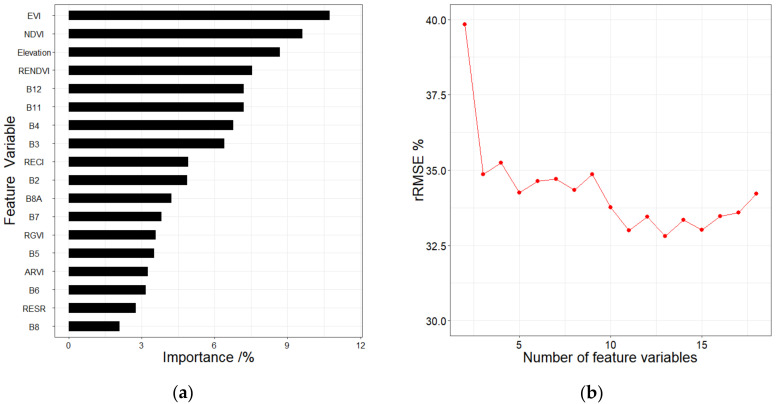
(**a**) The importance ranking of feature variables and (**b**) the random forest (RF) regression results (rRMSE) under different numbers of feature variables.

The random forest is based on the importance assessment of feature variables and error balance [48,49]. The importance is determined by their error contribution to the model. The Mean Decrease in Accuracy (MDA) is regarded as the indicator for measuring the importance of these feature variables [38,44]. The importance of all the feature variables was calculated and converted to percentages and by which the feature variables were ranked (Figure 4a). Multiple random forest regressions were established by gradually increasing the number of feature variables. When the relative root means square error (rRMSE) of the model reached the lowest, the variable combination formed by the number of variables was selected as the final result. Figure 4b shows that rRMSE reaches the minimum when the top 13 variables in the importance ranking form a variable combination.

**Figure 5:**
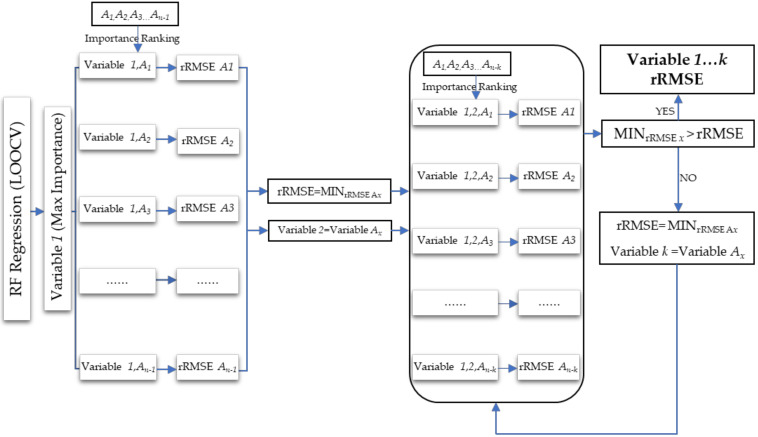
Feature variable selection process of the stepwise random forest (SRF) method.

**Figure 6:**
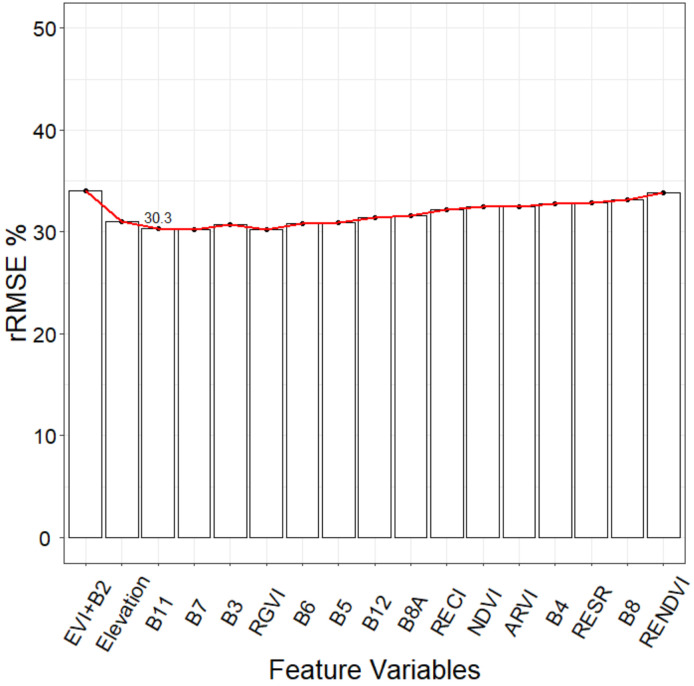
The SRF method applied to spectral variables for GSV estimation.

The RF method can evaluate the importance of a single feature variable but cannot directly provide the appropriate feature variable combinations. Different combinations of feature variables bring different rRMSE of RF regression, which can significantly affect the estimation accuracy. In order to select the appropriate combination of feature variables that can cause the smallest rRMSE, the stepwise random forest (SRF) was used to construct multiple random forest regression (RFR) models according to the importance ranking. Firstly, the feature variable with the highest importance was selected as a determining variable. Through the importance ranking, the variables were selected one by one to form the combinations with previously determined variables, and the RFR models were then respectively established. The corresponding variable combination with the smallest rRMSE was regarded as the newly determined variable combination, and the rRMSE was considered as the threshold. With the arrival of the next variable combination, if the new rRMSE was smaller than the threshold, the variable combination and threshold were then updated simultaneously. Until the variable combination and threshold were no longer updated, the variable combination with the minimum rRMSE was taken as the final selection result (Figure 5). Figure 6 shows that the lowest rRMSE of the feature variable combination consisting of EVI, B2, Elevation, B11, and B7 was 30.3%. The final feature variable combination was then used for GSV estimation. In addition, Boruta and VSURF are also used for variable selection. Finally, 16 and 3 variables are obtained for modeling and GSV estimation, respectively.

### 2.5. Extraction of the Texture Feature of Red-Edge Bands

The texture features can reveal the homogenous phenomenon in the image and reflect the arrangement characteristics of the surface features and structural organization of an object. Texture features have huge potential advantages for complex forest parameter estimation [47]. Due to the availability of multiple red-edge bands, the spectral information of Sentinel-2 images has proven to be very sensitive to vegetation parameters, but the effect of texture features on GSV estimation would still need to be verified. Gray-scale co-occurrence matrix (GLCM) has been widely used to extract texture information of remote sensing images [50]. In order to reduce the effect of different texture windows on texture feature values, eight texture features of red-edge bands were extracted through five texture windows (3 × 3, 5 × 5, 7 × 7, 9 × 9 and 11 × 11) [51,52,53,54]. Details of the extracted texture features are shown in Table 6.

**Figure 7:**
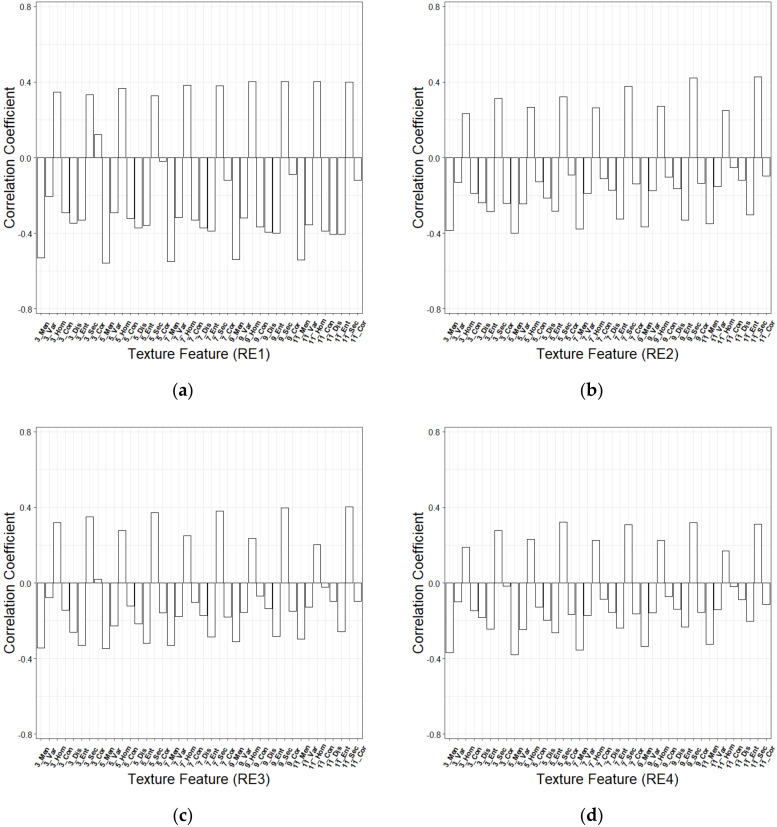
Correlation coefficients of texture features extracted from (**a**) RE1, (**b**) RE2, (**c**) RE3 and (**d**) RE4.

Each of the texture features was defined using the corresponding window size plus the red edge band and the texture metric. For example, 5 × 5_RE1_Men meant the mean texture measure from a window of 5 × 5 and band 5-Vegetation Red Edge 1. Correlation coefficients of all extracted texture features with GSV were calculated. It was found that the correlations of some texture features (e.g., 5 × 5_RE1_Men, 7 × 7_RE1_Men, 9 × 9_RE1_Men, and 11 × 11_RE1_Men) were higher than those of all the spectral variables. The distribution trends of correlation coefficients of the texture features extracted from the four red edge bands were similar. The overall correlation coefficient of the texture features in RE1 was relatively high, and the mean derived texture features under different texture windows provided the highest correlation coefficient (Figure 7).

**Figure 8:**
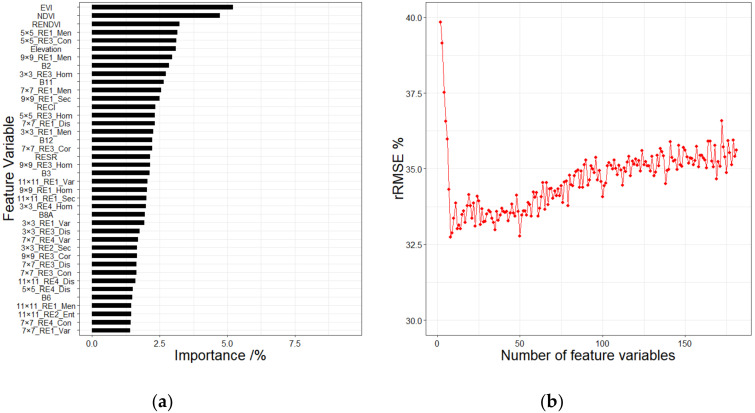
(**a**) The partial importance ranking of spectral variables, topographic factors, and texture features, (**b**) the change trend of rRMSE under different numbers of the feature variables.

**Figure 9:**
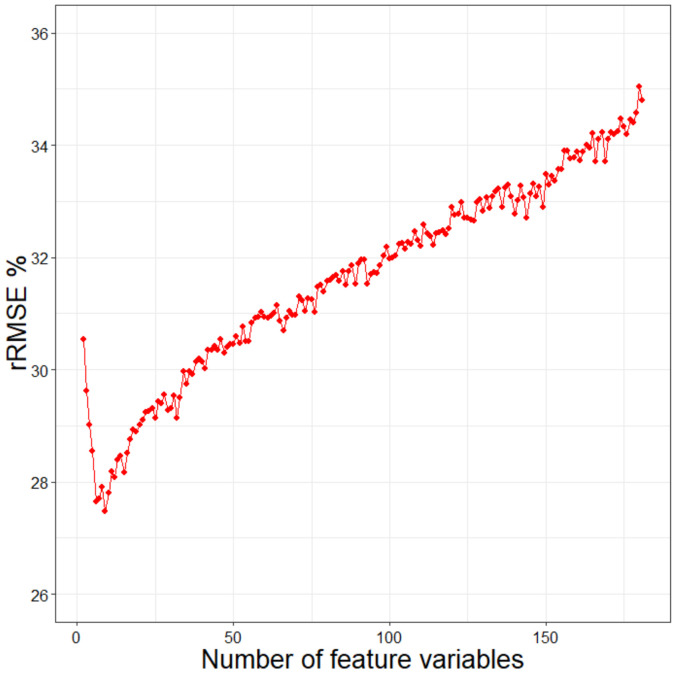
The change trend of rRMSE under the stepwise random forest method.

Together with the spectral variables and topographic factors, the importance of the texture features was evaluated. Figure 8 shows the partial importance ranking and the changing trend of errors under the RF method. Finally, a combination of eight variables with the minimum rRMSE was selected as the result. The SRF method was also used for the variable selection after adding the texture features. As the rRMSE started to decrease with the increase of the number of predictors, it finally reached the minimum when nine predictors were utilized and then continued to increase (Figure 9). With the participation of the texture features, the feature variable combinations were then selected by the LSR, RF, Boruta, VSURF, and SRF methods to establish the RFR models for estimating the GSV in the study area.

### 2.6. Random Forest Regression for GSV Estimation

As a non-parametric algorithm that is less sensitive to noise data, RF has been widely used in image classification and vegetation parameter estimation because it does not need the assumption of data distribution. Random forest regression can randomly generate a large number of regression trees used for estimation based on the used datasets and does not need to consider the collinearity between feature variables. It can effectively process large datasets and does not require reducing the dimension of high-dimensional data, which provides the potential for significantly improving its applicability [38,55]. Substantial research has shown that as a non-parametric algorithm, RF often performs better than the widely used parametric methods and thus has become more popular for estimation of vegetation parameters [36,37,55]. The *mtry* and *ntrees* are two important parameters that affect the model estimation accuracy. The *mtry* refers to the number of sample predictors of decision tree nodes, of which the default does not exceed the number of feature variables. And *ntrees* is the number of decision trees constructed by RF. Excessive *ntrees* will reduce the computing efficiency, and the final number can be determined based on the error change during the model construction [38]. In this study, RF modeling was therefore divided into participation with and without texture features, and then the parameter group results of the five variable combinations were determined respectively according to the error changes. The final prediction of each pixel depends on the average of the individual results of all the regression trees. The leave-one-out cross-validation (LOOCV) method [47] was applied in the RF modeling to assess the accuracy of all the predicted GSV values.

When no texture features were involved, the *mtry* of the five feature variable combinations were set as 2, 3, 1, 3, 2 and the *ntrees* were 150, 200, 200, 200 and 150, respectively. Similarly, the parameter groups (*mtry*, *ntrees*) with texture feature participation by five methods were finally determined as (2, 200), (2, 200), (7, 200), (2, 250) and (3, 200).

### 2.7. Accuracy Assessment of GSV Estimation

Using the GSV plot data and the feature variable combinations selected by the LSR, RF, Boruta, VSURF, and SRF methods, ten RFR models were developed to predict and map the GSV. These models were denoted as LSR-RFR, RF-RFR, and SRF-RFR. A LOOCV was used to assess the ten models [47]. The absolute residuals between the estimated values and the observations for the models were tested using the Student’s *t*-test. The estimation accuracy of the models was then evaluated by R^2^; Root Mean Squared Error (RMSE), rRMSE, the mean absolute error (MAE), and standard deviation of estimation (SD_e_) [56] as indicated in the formulae below

(1) $$R^{2}=1-\frac{\sum_{i=1}^{n}{\left(y_{i}-{\hat{y}}_{i}\right)}^{2}}{\sum_{i=1}^{n}{\left(y_{i}-\bar{y}\right)}^{2}}$$

(2) $$\mathrm{RMSE}=\sqrt{\frac{\sum_{i=1}^{n}{\left({\hat{y}}_{i}-y_{i}\right)}^{2}}{n}}$$

(3) $$\mathrm{rRMSE}=\frac{\sqrt{\frac{\sum_{i=1}^{n}{\left({\hat{y}}_{i}-y_{i}\right)}^{2}}{n}}}{\bar{y}}\times 100\%$$

(4) $$\mathrm{MAE}=\frac{1}{n}\sum_{i=1}^{N}\left|{\hat{y}}_{i}-y_{i}\right|$$

(5) $$SD_{e}=\sqrt{\frac{\sum_{i=1}^{n}{\left({\hat{y}}_{i}-\bar{y}\right)}^{2}}{n}}$$

where $y_{i}$ is the observed GSV, ${\hat{y}}_{i}$ is the estimated GSV based on RFR, $\bar{y}$ is the mean of the observed GSV, and n is the number of sample plots. All the models and calculations were done with the R 3.5.5 software.

## 3. Results

### 3.1. GSV Estimation and Mapping

Ten models (five RFR models with and without the texture features) were developed using the observed GSV combined with the feature variable combinations selected by the LSR, RF Boruta, VSURF, and SRF. The variable numbers without texture features in models were four, 13, 16, three, and five, while the numbers of variables with texture features were five, eight, 16, nine, and nine, respectively (Table 7). The estimated results represented in Table 7 shows that there was no significant difference between the estimation accuracy of LSR-RFR and RF-RFR. However, the SRF-RFR always achieved the best estimation performance whether there were texture features or not, which attained the highest determinant coefficient (R^2^ = 0.53 and 0.62) and the lowest RMSE, rRMSE, and MAE. The results implied that the SRF method achieved the best estimation effect, remaining statistically significant (Table 8). In addition, after the texture features participated in the modeling of the optimal SRF method, RMSE greatly decreased by 7.4%. In addition, the 95% confidence interval of SRF-RFR’ RMSE ranges from 46.64 to 56.06 m^3^/ha, and the RMSEs of other models do not fall within the range.

**Figure 10:**
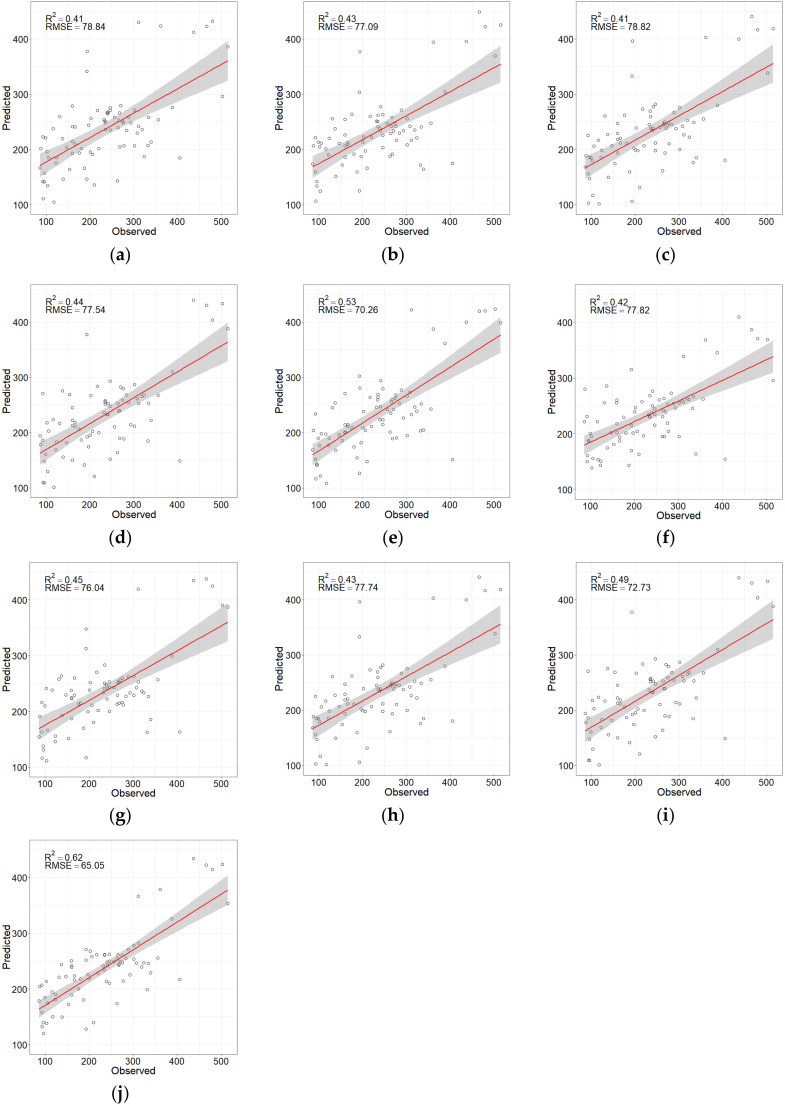
Scatter plots of the observed and predicted GSV values by (**a**) LSR-RFR without texture features, (**b**) RF-RFR without texture features, (**c**) Boruta-RFR without texture features, (**d**) VSURF-RFR without texture features, (**e**) SRF-RFR without texture features, (**f**) LSR-RFR with texture features, (**g**) RF-RFR with texture features, (**h**) Boruta-RFR with texture features, (**i**) VSURF-RFR with texture features, and (**j**) SRF-RFR with texture features.

The scatter plots in Figure 10 show the fitting between the observed GSV and the GSV predicted by the ten models. The fitting effect of the ten models is similar, having both overestimation and underestimation values. Among them, the SRF-RFR shows to be the best fit. While the estimated values are basically distributed on both sides of the fitting line, there are also some overestimation values. Compared with the one without the texture features, the model SRF-RFR with the texture features greatly reduced the overestimations and underestimations.

**Figure 11:**
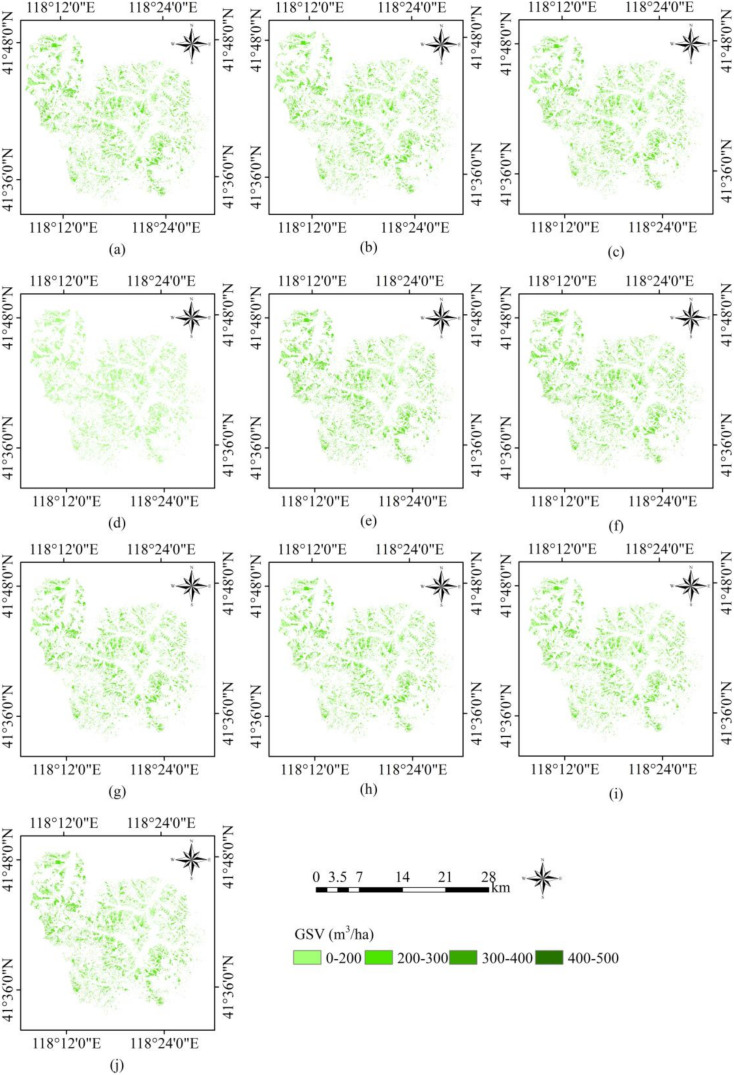
Spatial distributions of GSV estimates in the Wangyedian forest farm predicted by (**a**) LSR-RFR without texture features, (**b**) RF-RFR without texture features, (**c**) Boruta-RFR without texture features, (**d**) VSURF-RFR without texture features, (**e**) SRF-RFR without texture features, (**f**) LSR-RFR with texture features, (**g**) RF-RFR with texture features, (**h**) Boruta-RFR with texture features, (**i**) VSURF-RFR with texture features, and (**j**) SRF-RFR with texture features.

The ten RFR models were separately used for mapping the GSV of the Wangyedian forest farm and led to similar spatial distributions of GSV (Figure 11). The GSV spatial distribution by the SRF-RFR model with the texture features best agrees with the actual forest distribution. The GSV values of 300–500 m^3^/ha are mainly distributed in the northwest and west of the forest farm. The eastern and central areas are mainly farmlands and built areas where the distribution of GSV is negligible.

### 3.2. Uncertainty Analysis

In order to evaluate the adaptability of the SRF-RFR, the following two methods were used to analyze the uncertainty of the residuals generated by the SRF-RFR: (1) the significance of the relationship between the feature variables and the residuals; and (2) the comparison of estimates between the separate sample plot sets by tree species and the pooled sample plot set.

Our results of the correlation analysis show that there were positive and negative correlations found between the feature variables and residuals. The feature variable that possesses the highest correlation with residuals is ‘Elevation’ with a Pearson correlation coefficient of −0.17 (*p* > 0.05). The absolute values of all the correlations were similarly low, and not statistically significant (Table 9). In addition, the VIF values of variables determined by SRF were very low, which further improves the reliability of the model and SRF method.

**Figure 12:**
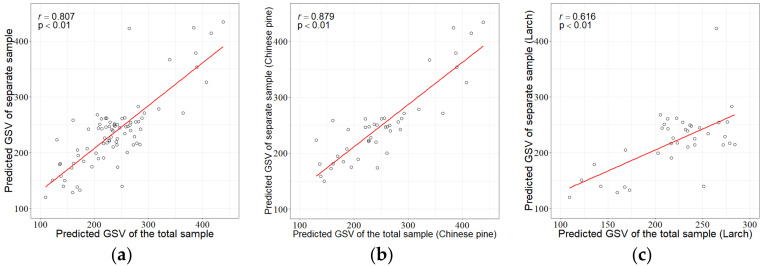
Scatter plots of the GSV predictions by the total or pooled sample plot set against the GSV predictions by separate sample plot sets of tree species: (**a**) all the tree species, (**b**) Chinese pine, and (**c**) Larch.

The feature variables show dissimilar importance in different sample sizes. Ranking of the feature variables was separately conducted under the Chinese pine and larch sample plot sets, and the SRF method was used to determine the final feature variable combination to establish the RFR for GSV estimation. The results show that the selected feature variables of the Chinese pine were basically the same as those for the total or pooled sample plot set; however, the results for larch were quite different from those for the total sample plot set. The correlation coefficients between the predicted GSV values from the pooled sample plot and the separate sample plot sets were 0.807 (*p* < 0.01) for the overall, 0.879 (*p* < 0.01) for Chinese pine, and 0.616 (*p* < 0.01) for larch (Figure 12). There were significant correlations for the three groups, which indicate that it is acceptable to estimate GSV whether or not the separation of the sample plots by tree species is made. The estimation accuracies from the pooled sample plot set and the separate sample plot sets by tree species were compared in Table 10. Chinese pine provided more accurate estimations with smaller rRMSE values of 25.4% and 24.9% compared to the corresponding values of larch, 31.3%, and 29.8% for the pooled sample plot set and the species separate sample plot sets, respectively.

## 4. Discussion

### 4.1. Feature Variable Selection Method

Different combinations of feature variables will lead to different modeling accuracies. Choosing the appropriate variable selection method can significantly improve the GSV estimation. Original spectral bands, VIs, and topographic factors are commonly used feature variables for GSV estimation [39,57]. The predictors significantly correlated with GSV were extracted in our study and formed the feature variable combinations by five selection methods. It was found that the LSR method provided the best linear combination of feature variables, but it requires variables without collinearity. However, the relationships between GSV and feature variables may not be linear, likely due to the complexity of forest ecosystems, which limits the estimation accuracy of the model [46]. The RF method can evaluate the importance of feature variables based on non-linear relationships, allowing for the selection of feature variables with high importance for modeling [28,38]. Xie et al. [58] used an RF algorithm to measure the importance of all candidate feature variables and then selected the predictors for regional GSV prediction and mapping, and the obtained results were acceptable (R^2^ = 0.618). This implies that RF algorithms can be used to select robust and stable predictors based on importance measures. The Boruta and VSURF methods [31] were compared to the SRF method. And the RMSEs obtained by the two methods were 77.74 m^3^/ha and 72.73 m^3^/ha, respectively, which were increased by 16.3% and 10.6% compared with SRF (*p* < 0.001). In addition, the two methods were tested in time costing, which spent 78s and 297s in 181 variables (with texture features), respectively. However, the SRF method with higher accuracy takes 89s. Compared with Boruta, the time is not significantly increased, but the accuracy of 16.3% is improved, which makes the time cost worth it.

While different from the LSR method based on linear correlation, the RF method selects variables based on their importance rankings, thus improving the selection process. However, the importance assessment is relative, where the importance of a single variable in different combinations of feature variables can also vary [32,38]. In response, we proposed the SRF method for the selection of appropriate feature variable combinations. In this method, we first used the importance ranking determined by the whole sample as a reference and then selected the appropriate combination according to the error change as the number of feature variables increased. Finally, we found that the RFR with the variable combination determined by the SRF method had the highest estimation accuracy. As a result, SRF can effectively reduce the estimation error by establishing multiple RFR models based on importance ranking and comparing the results from different variable combinations.

### 4.2. Uncertainty Analysis

Through the comparison of five feature variable selection methods combined with the RF model, a map showing the most accurate estimates of GSV for the planted coniferous forests in Wangyedian Forest Farm was produced. However, we found that the estimates were associated with uncertainties. The uncertainty of GSV estimates often results from atmospheric effects, sensor effects, sample plot GSV measurement errors, feature variable selection, and estimation model [27,59]. At present, the atmospheric effects and sensor errors cannot be completely eliminated [57,59]. Also, there was a substantial variation of the GSV among the training sample plots. This suggests that the sampling design and sample size could be better improved in prospective studies.

Collecting tree-by-tree measurements requires a sampling design to represent the average level of forest cover in a given study area. Accurate measurements and the selection of appropriate volume equations can greatly determine the acquisition of true volume values [59]. Deforestation, forest fires, and natural regeneration can all affect the applicability of the volume formula [27]. It is therefore crucial for the forest survey program in Wangyedian Forest Farm to obtain updated forest resource data annually while checking and calibrating volume equations periodically [9,27]. This would help to substantially reduce the error of the plot level GSV estimates.

It was found that the combination of feature variables directly affects the accuracy of model estimation [38,57]. Too many variables tend to increase model errors and program running time. Therefore, selecting the appropriate feature variables can significantly improve the efficiency of modeling and prediction [32,38]. The commonly used LSR method is sensitive to the linear model, but the model itself is limited in GSV estimation given the complex forest structure [51]. The RF can evaluate the importance of feature variables, which helps to efficiently select the appropriate feature variables [38,49,51]. Compared with the LSR, the RF showed significant improvement to the model given that feature variables were selected according to their importance ranking. While RF can only provide the importance of a single feature variable, the importance of the same feature variable may change in different combinations of the variables [32]. On the basis of primary importance ranking, variable combinations should be considered to extract significant combinations of the variables. The SRF provides the potential to select the combinations of feature variables and can lead to the smallest error in GSV estimation. All the variable combinations selected by SRF can be tested for their significance with absolute residuals produced by the models. The variable combinations that have no significant contribution to the reduction of residual error can be eliminated. Thus, SRF provides a powerful tool for the selection of predictors and improvement of estimation accuracy.

Linear models are easy to realize and understand. However, their estimation accuracy of forest parameters in complex forest ecosystems is limited [51]. In contrast, non-parametric methods can model non-linear relationships and result in more accurate results in GSV estimation [27,35,57,58,59]. In our analysis, the SRF-RFR achieved the minimum rRMSE of 28.0% in the GSV estimation of Wangyedian forests, while the LSR-RFR, RF-RFR, Boruta-RFR, and VSURF-RFR resulted in the rRMSE values of 33.5%, 32.7%, 33.5%, and 31.3%, respectively. The RMSE obtained by SRF-RFR was 16.4%, 14.4%, 16.3%, and 10.6% smaller than those by LSR-RFR, RF-RFR, Boruta-RFR, and VSURF-RFR. Compared with the pooled sample plot set moreover, using the separate sample plot sets by species for the development of SRF-RFR models slightly increased the estimation accuracy of the GSV. The reason might be because separating the sample plots by tree species decreased the variation of the within model GSV. Due to the limited space, this study only dealt with the improvement of RF. In future studies, other non-parametric methods such as *k*-nearest neighbors (*k*NN), support vector machine (SVM), and artificial neural network (ANN) should be considered [59,60].

## 5. Conclusions

Accurate estimation of GSV is crucial for regional and global forest resource assessment and ecosystem dynamic monitoring. This study proposed the SRF, an improved RF selection method of feature variables, and compared its results with those from four widely used methods (LSR, RF, Boruta, and VSURF) for GSV estimation using Sentinel-2 and observed GSV data for the Wangyedian forest farm. The following conclusions were drawn: (1) The red-edge bands of Sentinel-2 images have more significant correlations with GSV than other used feature variables, and the red-edge bands derived feature variables have priority in terms of their contribution to the reduction of model errors. Introducing the red edge features into the GSV models greatly improves the estimation accuracy of the GSV; and (2) Compared with the LSR, RF, Boruta, and VSURF methods, the SRF performed best in the selection of feature variables, and the SRF-RFR led to the smallest rRMSE of the GSV predictions. Compared with the LSR-RFR, RF-RFR, Boruta-RFR and VSURF-RFR, the SRF-RFR model reduced the RMSE by 16.4%, 14.4%, 16.3% and 10.6%, respectively. Thus, the SRF-RFR method offers the potential for improving the estimation accuracy of the GSV and provides a reference for forest dynamic monitoring.

## Figures and Tables

**Table 1:** Summary statistics of per unit growing stem volume (GSV) values (m^3^/ha).

Tree Specie	Plot Number	Range of Values	Mean	Standard Deviation	Coefficient of Variation (%)
Chinese pine	43	91.97–514.96	245.52	110.59	45.0
Larch	38	86.17–466.23	217.21	91.84	42.3
Total	81	86.17–514.96	232.24	102.59	44.2

**Table 2:** Information of the acquired Sentinel-2 data.

Image Identification	Product Level	Acquisition Date
S2A_MSIL1C_20170922T025541_N0205_R032_T50TNM_20170922T030440	L1C	22 September, 2017
S2A_MSIL1C_20170922T025541_N0205_R032_T50TPM_20170922T030440	L1C	22 September, 2017

L1C: Level-1C

**Table 3:** Bands of the Sentinel-2 images used in the study.

Sentinel-2 Bands	Central Wavelength (nm)	Bandwidth (nm)	Spatial Resolution (m)
Band 2-Blue	496.6	98	10
Band 3-Green	560.0	45	10
Band 4-Red	664.5	38	10
Band 5-Vegetation Red Edge	703.9	19	20
Band 6-Vegetation Red Edge	740.2	18	20
Band 7-Vegetation Red Edge	782.5	28	20
Band 8-NIR	835.1	145	10
Band 8A-Vegetation Red Edge	864.8	33	20
Band 11-SWIR	1613.7	143	20
Band 12-SWIR	2202.4	242	20

**Table 4:** Spectral variables and topographic factors used in this study.

Feature Variable	Description	Reference
Band Reflectivity	B2-BLUE, B3-GREEN, B4-RED, B5-Red Edge1, B6-Red Edge2, B7-Red Edge3, B8-NIR, B8A-Red Edge4, B11-SWIR1, B12-SWIR2	[39]
Vegetation Index	Normalized Difference Vegetation index (NDVI)	[39]
	Enhanced Vegetation index (EVI)	[39]
	Red-green vegetation index (RGVI)	[39]
	Atmospherically resistant vegetation index (ARVI)	[39]
	Red Edge Normalized Difference Vegetation index (RENDVI)	[44]
	Red Edge Chlorophyll Index (RECI)	[45]
	Red Edge Simple Ratio (RESR)	[45]
Topographic Factor	Elevation	[46]
	Slope	[46]
	Aspect	[46]

**Table 5:** Statistical results of the linear stepwise regression (LSR) method.

Variable	Coefficient	Significance	VIF
Constant	−125.48	0.03	-
B7	−1162.73	0.00	1.10
Elevation	0.29	0.00	1.07
RGVI	−111.09	0.04	1.60
RENDVI	379.13	0.00	1.65

VIF: variance inflation factor.

**Table 6:** Texture features extracted under different texture windows.

Texture Window	Red Edge Band	Texture Metric
3 × 3, 5 × 5, 7 × 7, 9 × 9, 11 × 11	Band 5-Vegetation Red Edge 1 (RE1), Band 6-Vegetation Red Edge 2 (RE2), Band 7-Vegetation Red Edge 3 (RE3), Band 8A-Vegetation Red Edge 4 (RE4)	Mean (Men)
		Variance (Var)
		Homogeneity (Hom)
		Contrast (Con)
		Dissimilarity (Dis)
		Entropy (Ent)
		Second moment (Sec)
		Correlation (Cor)

**Table 7:** Accuracy comparison of the GSV estimates (m^3^/ha) from the RFR models with various combinations selected by the LSR, RF, Boruta, VSURF, and SRF variable selection methods.

Model	Method	Variables Combination	R^2^	RMSE	rRMSE (%)	MAE	SDe
Without texture features	LSR	B7, Elevation, RENDVI, RGVI	0.41	78.84	33.9	62.17	70.95
	RF	EVI, NDVI, Elevation, RENDVI, B12, B11, B4, B3, RECI, B2, B8A, B7, RGVI	0.43	77.09	33.2	61.13	72.27
	Boruta	EVI, NDVI, Elevation, RENDVI, B12, B11, B4, B3, RECI, B2, B8A, B7, RGVI, B5, B6, RESR	0.41	78.72	33.9	63.06	73.43
	VSURF	EVI, NDVI, B2	0.44	77.54	33.4	59.90	80.73
	SRF	EVI, B2, Elevation, B11, B7	0.53	70.26	30.3	56.06	71.35
With texture features	LSR	5 × 5_RE1_Men, Elevation, 11 × 11_RE2_Sec, 11 × 11_RE2_Ent, 9 × 9_RE2_Con, B7, 9 × 9_RE3_Cor	0.42	77.82	33.5	59.04	58.18
	Boruta	NDVI, EVI, B11, B12, B2, B3, B4, Elevation, RECI, RENDVI, 3 × 3_RE1_Men, 5 × 5_RE1_Men, 7 × 7_RE1_Men, 9 × 9_RE1_Men, 11 × 11_RE1_Men, 3 × 3_RE3_Hom	0.43	77.74	33.5	61.34	72.34
	RF	EVI, NDVI, RENDVI, 5 × 5_RE1_Men, 5 × 5_RE3_Con, Elevation, 9 × 9_RE1_Men, B2	0.45	76.04	32.7	59.67	73.96
	VSURF	EVI, NDVI, 5 × 5_RE1_Men, 9 × 9_RE1_Men, B12, Elevation, B2, B3, 9 × 9_RE1_Sec	0.49	72.73	31.3	58.26	73.59
	SRF	EVI, 11 × 11_RE1_Sec, B2, 5 × 5_RE1_Sec, Elevation, 11 × 11_RE3_Ent, 3 × 3_RE3_Var, 3 × 3_RE1_Hom, 9 × 9_RE4_Var	0.62	65.05	28.0	52.69	65.04

**Table 8:** The test results (*p*-values) of significant differences among the models based on the absolute residuals between the estimated and observed GSV values using student’s *t* test.

Model	Variable Selection Method	LSR	RF	Boruta	VSURF
Without texture features	LSR				
	RF	−7.86 (0.00)			
	Boruta	−0.42 (0.67)	7.84 (0.00)		
	VSURF	0.51 (0.61)	8.38 (0.00)	0.77 (0.44)	
	SRF	2.01 (0.04)	8.63 (0.00)	2.63 (0.01)	2.13 (0.00)
With texture features	LSR				
	RF	0.08 (0.93)			
	Boruta	−0.33 (0.74)	−0.82 (0.41)		
	VSURF	0.42 (0.66)	0.65 (0.52)	1.82 (0.07)	
	SRF	2.01 (0.04)	2.40 (0.01)	2.66 (0.00)	2.00 (0.04)

**Table 9:** The Pearson correlation coefficients of the residuals with the associated feature variables.

Study Area	Feature Variable	Residual	VIF
Wangyedian Forest Farm	EVI	−0.09	3.222
	11 × 11_RE1_Sec	−0.04	6.270
	B2	0.12	3.244
	5 × 5_RE1_Sec	0.01	5.308
	Elevation	−0.17	1.064
	11 × 11_RE3_Ent	−0.04	6.115
	3 × 3_RE3_Var	−0.13	1.568
	3 × 3_RE1_Hom	−0.08	2.768
	9 × 9_RE4_Var	0.04	2.430

**Table 10:** Feature variable results based on SRF method under two tree species (m^3^/ha).

Sample Size	Tree Specie	R^2^	RMSE	rRMSE (%)	MAE
Total or pooled sample plot set	Chinese pine	0.73	62.40	25.4	51.05
	Larch	0.44	67.92	31.3	54.53
	Total	0.62	65.05	28.0	56.69
Separate sample plot sets by species	Chinese pine	0.68	63.20	24.9	50.06
	Larch	0.53	65.67	29.8	49.45
	Total	0.62	64.37	27.7	49.77
